# Comprehensive Application of Graphene: Emphasis on Biomedical Concerns

**DOI:** 10.1007/s40820-019-0237-5

**Published:** 2019-01-12

**Authors:** S. Syama, P. V. Mohanan

**Affiliations:** Toxicology Division, Biomedical Technology Wing, Sree Chitra Tirunal Institute for Medical Sciences and Technology, Thiruvananthapuram, Kerala 695 012 India

**Keywords:** Graphene, Biomedical, Bioprinting, Toxicity, Photothermal therapy

## Abstract

Graphene, *sp*^2^ hybridized carbon framework of one atom thickness, is reputed as the strongest material to date. It has marked its impact in manifold applications including electronics, sensors, composites, and catalysis. Current state-of-the-art graphene research revolves around its biomedical applications. The two-dimensional (2D) planar structure of graphene provides a large surface area for loading drugs/biomolecules and the possibility of conjugating fluorescent dyes for bioimaging. The high near-infrared absorbance makes graphene ideal for photothermal therapy. Henceforth, graphene turns out to be a reliable multifunctional material for use in diagnosis and treatment. It exhibits antibacterial property by directly interacting with the cell membrane. Potential application of graphene as a scaffold for the attachment and proliferation of stem cells and neuronal cells is captivating in a tissue regeneration scenario. Fabrication of 2D graphene into a 3D structure is made possible with the help of 3D printing, a revolutionary technology having promising applications in tissue and organ engineering. However, apart from its advantageous application scope, use of graphene raises toxicity concerns. Several reports have confirmed the potential toxicity of graphene and its derivatives, and the inconsistency may be due to the lack of standardized consensus protocols. The present review focuses on the hidden facts of graphene and its biomedical application, with special emphasis on drug delivery, biosensing, bioimaging, antibacterial, tissue engineering, and 3D printing applications.

## Highlights

- The introduction of graphene will certainly uncover new advanced materials, and many more future technologies will become realistic in the forthcoming years.
- The present review article includes more recent publications about the biomedical application and cellular interaction of graphene. It is also updated with modern approaches such as use of graphene inks for 3D printing application.
- Moreover, the importance of protein corona in modulating the cellular interaction, which was overlooked in previous review publications, is also included in this article.
- The possible biological outcomes and toxicity when graphene is exposed to living organisms at the cellular and organ level are explained.

## Graphical abstract

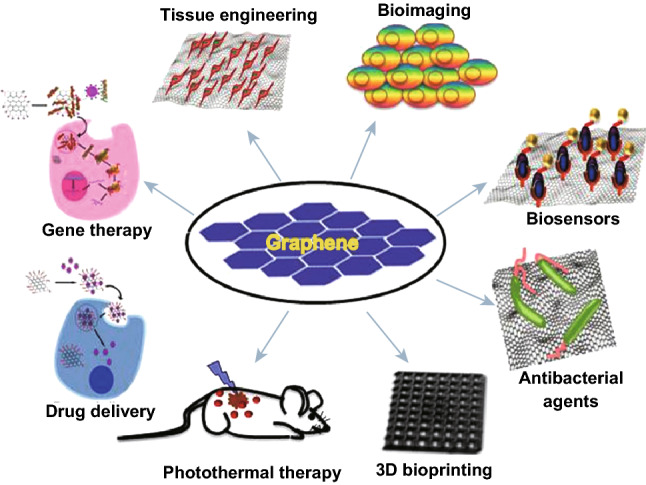

## Introduction

Graphene, the basic structure for all graphitic materials, is a two-dimensional (2D) sheet of carbon atoms arranged in a honeycomb lattice structure. The outstanding properties of graphene including its mechanical strength, electrical conductivity, thermal conductivity, and large surface area highlight its widespread applications. However, in reality, the hydrophobic nature of graphene impedes its use in the biomedical field [1]. Graphene derivatives such as pristine (nonoxidized) graphene sheets, graphene oxide (GO), and reduced graphene oxide (RGO) have been extensively studied for various biomedical applications. Pristine graphene sheets are less advantageous than GO and RGO owing to their hydrophobicity and inability to form stable homogenous dispersions. Hence, the highly oxidized form (GO) and the less oxidized form (RGO) of graphene are the most widely studied because of the ease of manipulation for obtaining a more stable aqueous suspension with intrinsic properties.

Among the graphene family materials, GO, a derivative of graphene with oxygen-containing functional groups, is widely recommended for biomedical use as it is readily soluble in water (hydrophilic). GO possesses carboxyl, epoxyl, and hydroxyl functional groups distributed throughout the basal plane and its edges. The stronger hydrogen bond interaction between the oxygen molecule of the epoxide groups of GO and water molecules maintains its stacked structure [2].

Graphite oxide (GtO), another form of graphene, differs from GO in its structure, but, chemically, the two are similar. GtO is a highly stacked structure with oxide functionalities, whereas GO has a wide spacing between the layers because of water intercalation [3]. The structure of GO can be explained by the Lerf–Klinowski model [4] as a hexagonal carbon lattice with a hydroxyl and an epoxyl group on the plane and a carboxyl and a carbonyl group on the edges. The covalent C–O bond disrupts the *sp*^2^ conjugation of the lattice, making GO an insulator [5]. The electronic and mechanical properties of GO can be modified by controlling the rate of oxidation. GO may achieve different conformations in aqueous solution (folding, bending, scrolling, and planar structures); the presence of both hydrophilic and hydrophobic domains promotes bending of sheets, which is the most stable conformation form. Graphene is obtained from GO using thermal annealing or chemical-reducing agents. The chemically derived graphene sheets are also called reduced graphene oxide (RGO). The reduction of GO using different reducing agents such as hydrazine hydrate [6], hydroquinone [7], ascorbic acid [8], sodium borohydride [9], and strong alkaline solutions [10, 11] has been reported. The most commonly used one is hydrazine [12] because of its nonreactivity toward water. The reduction of GO leads to loss of functional groups on its surface, resulting in increased hydrophobicity. The surface area of RGO decreases because of aggregation or precipitation during reduction and also because of incomplete exfoliation. The size, shape, surface functionalities, lateral dimension, state of oxidation, agglomeration, and presence of contaminants affect the underlying biological response toward graphene. Hence, controlled synthesis should be carried out to transfer a graphene material from the laboratory to the clinic for various biological applications. The present review provides a detailed outlook and added information on the different methods of graphene synthesis, its biomedical applications, and the major biological consequences imposed by graphene-derived materials. In addition, the present review also discusses in detail the importance of protein corona, which modulates the cellular interaction. This topic was overlooked in previous review publications. The adverse biological outcomes and toxicity at the cellular and organ level, when graphene is exposed to living organisms, are also explained in detail.

## Methods of Graphene Synthesis

Despite the tremendous increase in the number of literature studies on graphene synthesis, the large-scale industrial production of graphene is still difficult to achieve because of the various synthetic methods adopted, which vary with their application. Graphene is obtained from GO by removing oxygen-containing functional groups via simple chemical or thermal reduction. Basically, graphene synthesis is categorized into two types: a top-down and a bottom-up approach. The top-down approach uses chemical ablation, electrochemical oxidation, or plasma treatment to cut down larger graphene sheets into smaller pieces. In contrast, the bottom-up method involves building up larger graphene sheets from simple carbon precursors.

### Synthesis of Graphene from Graphite

#### Mechanical Exfoliation of Graphite

Graphite, an allotrope of carbon, is a naturally available chemical having numerous defects in its structure. It is the precursor material for the synthesis of different forms of graphene. Mechanical exfoliation of graphene from pyrolytic graphite using Scotch tape was developed by Geim and Novoselov [12]. The advantage of this method is that it maintains the structural integrity of graphene sheets, whereas the disadvantages are the uncontrollable size and thickness of the resulting graphene sheets and that this method cannot be extrapolated for large-scale synthesis either.

#### Chemical Exfoliation of Graphite

##### Synthesis of GO from Graphite

**Fig. 1:**
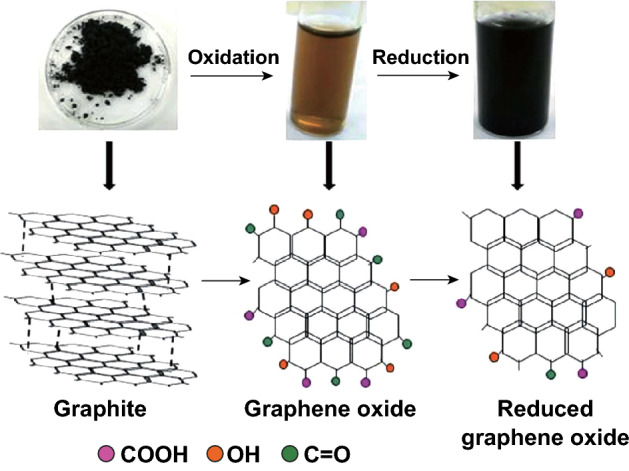
Process of graphene synthesis. Graphite is exfoliated and oxidized to form hydrophilic graphene oxide (brown solution). Graphene oxide is further reduced to obtain reduced graphene oxide, which is less stable in water (black solution)

Chemical exfoliation of graphite remains a simple, efficient, and cost-effective method for producing hydrophilic GO (Fig. 1). The properties of the synthesized graphene vary depending on the source of graphite, the oxidant used, and the reaction conditions followed. In this method, GtO is synthesized from graphite powders under chemical oxidation. The primary route for the synthesis of GtO was developed by Hummer and Offeman by mixing graphite in a mixture of potassium permanganate and sulfuric acid [13]. The reaction of KMnO_4_ with concentrated H_2_SO_4_ gives explosive Mn_2_O_7_ (manganese heptoxide), which oxidizes graphite [14].

$$2{\text{KMnO}}_{4} + 2{\text{H}}_{2} {\text{SO}}_{4} \to {\text{Mn}}_{2} {\text{O}}_{7} + {\text{H}}_{2} {\text{O}} + 2{\text{KHSO}}_{4}$$

Oxidation of the defect sites of graphite can be initiated using different oxidizing agents such as nitric acid, potassium chlorate [15], and potassium permanganate. Oxidation of graphite breaks the *sp*^2^-hybridized carbon sheets into a graphitic *sp*^2^ domain surrounded by oxidized *sp*^3^ domains and several defects [16]. The oxidized graphite (GtO) is a stacked structure similar to that of graphite but has wider spacing and several oxygen-containing functional groups distributed in the sheets. Further exfoliation of GtO results in the formation of a single layer or a few layers of GO. GtO is exfoliated in water using mechanical force (sonication/centrifugation) to separate the stacked structure. The exfoliation of graphite increases the interplanar space by intercalating oxygen moieties in-between the sheets, thereby weakening the interactions between carbon planes [17].

GO contains chemically reactive functional groups such as carboxylic acid on the edges and hydroxyl and epoxy groups on the basal plane. These oxygen-containing functional groups are modified to formulate biocompatible graphene for clinical use. The carboxylic acid groups are activated by various chemicals such as thionyl chloride (SOCl_2_), 1-ethyl-3-(3-dimethylaminopropyl)-carbodiimide, and *N*,*N*′-dicyclohexyl-carbodiimide, followed by the addition of amines or hydroxyl-forming covalent amide or ester bonds. In addition, polymers can also be grafted on the carboxylic end of GO to make it dispersible in water and solvents. Functionalization of the epoxy groups involves the ring-opening reaction [18] in which the amine groups attack the α-carbon. Noncovalent functionalization of GO and RGO is also possible via *π*–*π* stacking and van der Waals interaction. Preparation of stable dispersions of graphene remains an unsolved problem because of its hydrophobic nature. This can be achieved by sonicating graphene suspension for several hours and also by using surfactants or polymers. Details of the effect of different solvents and surfactants and the importance of sonication to obtain a stable aqueous dispersion of GO were well thoroughly investigated by Khan et al. [19]. In their study, GO was dispersed in solvents such as water, tetrahydrofuran (THF), *N*,*N*-dimethylformamide (DMF), ethylene glycol, acetone, pyridine, 2-propanol, methanol, ethanol, and dimethyl sulfoxide (DMSO), and was sonicated for 400 h along with the addition of sodium cholate. The treated GO samples were stable in water, DMF, ethylene glycol, and pyridine, and remained as homogenous dispersion even after 3 weeks without any aggregation. Similarly, Paredes et al. [20] also reported the dispersion behavior of GtO in various solvents (water, acetone, ethanol, methanol, 1-propanol, ethylene glycol, DMSO, DMF, *N*-methylpyrrolidone (NMP), pyridine, THF, dichloromethane, *o*-xylene, and *n*-hexane) following 1 h of bath sonication. Immediately after sonication, the GtO remained dispersed in almost all the solvents. However, long-term stability (3 weeks) was achieved only in water, ethylene glycol, DMF, NMP, and THF. Both these studies identified suitable solvents to improve GO dispersibility for further application. Simple magnetic stirring and a heating method were also demonstrated for the synthesis of aqueous dispersion of single-layer GO [21]. Nanosized GO was obtained by ultrasonication of GO, which resulted in fragmentation from defect regions followed by the elimination of oxygen-containing functional groups at hot spots [22].

##### Chemical Reduction of GO to RGO

The reduction of GO under physical (temperature) or chemical (reducing agents) reduction conditions results in the formation of RGO. The chemically reduced graphene sheet undergoes incomplete reduction, leaving behind a few oxygen-containing functional groups. Chemically reduced graphene looks similar to pristine graphene in terms of electrical, thermal, and mechanical properties. Commonly followed hydrazine-based reduction increases the presence of *sp*^2^ domains on graphene sheets. Other reducing agents such as sodium borohydride [9], hydroquinone [7], gaseous hydrogen [23], strong alkaline solutions [10, 11], and ascorbic acid [8] are also employed for the reduction of GO. The disadvantage of using chemicals for the reduction process is the presence of impurities in the final product, which are sometimes difficult to remove. Yet, chemical reduction of GO is the most favorable method because of its high yield for large-scale applications.

##### Thermal Reduction of GO to RGO

GO is also treated at higher temperatures to exfoliate its stacked structure. This physical method of reduction extrudes CO_2_ gas, which creates enough pressure to separate the stacked structures [24]. However, the thermal reduction creates structural defects on the surface of GO, which also affects the electronic properties of graphene materials [18].

##### Electrochemical Reduction of GO to RGO

Electrochemical reduction of GO was developed to avoid the use of harmful reductants such as hydrazine. In this method, GO films are deposited on the surface of various substrates; after placing electrodes on the opposite end of the film, voltammetry is run [25]. The exact mechanism of reduction remains unknown, although it is suggested that hydrogen ions present in the buffer solution are responsible for the reduction. The main drawback of electrochemical reduction is its scalability. Moreover, there is a possibility that the deposited RGO on the electrode surface hinders further reduction. Chemical vapor deposition (CVD) is one of the most feasible and inexpensive methods for synthesizing single- or multilayer graphene sheets. Here, graphene sheets are grown on the surface of transition metals such as Ni, Cu, and Pd that act as catalysts [26]. Hydrocarbon gases heated at 1000 °C decompose into atomic radicals and dissolute into Ni, which is segregated and crystallized to form graphene. Graphene sheets synthesized from CVD undergo expansion when the temperature decreases, whereas the metal catalyst shrinks, resulting in the formation of ripples. These defective structures (ripples) are more reactive and are an important factor for the bioconjugation of graphene. From the metal surface, graphene sheets must be transferred to insulating materials to fabricate electronic devices. This can be achieved by either chemical or thermal etching of graphene sheets from the substrate.

##### Green Reduction of GO to RGO

A green route for graphene synthesis under alkaline conditions was reported [10]. The method involves the addition of NaOH or KOH to exfoliate GO solution at high temperatures. Graphite oxidizes in the presence of strong acids to form GO. The principle behind this method is the reversible deoxygenation of GO in the presence of alkaline agents. The reaction occurs faster at increased pH. In addition, the incomplete removal of oxygen groups under this condition (high pH) makes the negatively charged graphene sheets repulsive and prevents agglomeration. Green reduction of GO using amino acids [27], tea solution [28, 29], melatonin [30], glucose [31], reducing sugar [32], bovine serum albumin (BSA) [33], bacteria [34], and plant-derived phytochemicals [35] to reduce the detrimental effects of using toxic reductants has been reported.

### Synthesis of Graphene from GtO

Exfoliation and reduction of sulfuric acid-intercalated GtO at > 100 °C resulted in single-layer graphene sheets of 1.07 µm mean diameter following dispersion in DMF [36]. When graphite is oxidized in the presence of sulfuric acid, the sulfuric acid molecules intercalate between the sheets and reacts with intercalated sulfuric acid (ISA) to form sulfuric acid-intercalated GtO. ISA catalyzes dehydration (removal of O and H atoms), generating high-pressure steam or heat that decomposes the carbonyl groups. The graphene sheets produced by this process are highly conductive and possess fewer defects and lower oxygen content.

Microwave-assisted reduction, ion bombardment, hydrazine-free aqueous route, ultraviolet irradiation, thermal reduction, chemical reduction, electrochemical reduction, and solvothermal reduction have been reported in literature [37]. In thermal reduction, rapid heating at high temperatures (1000–2000 °C) exfoliates GtO sheets into graphene. The sudden increase in temperature decomposes the oxygen-containing functional groups into gases, which creates enough pressure to separate stacked layers. However, the elevated temperature can cause structural damage to the graphene sheets and also affect its electronic properties. Dao et al. [38] utilized thermal reduction (1100 °C) to obtain highly oxidized GtO in a small size. It was stated that the size of the starting material graphite will affect the size, chemical structure, degree of oxidation, and other properties of the resultant GtO. Vacuum-assisted microwave reduction was reported to yield graphene with high C/O ratio and partial hydrogenation [39]. Microwave irradiation under a vacuum leads to outgassing and plasma formation. The plasma helps in the uniform distribution of heat from microwave radiation and also promotes hydrogenation. Further evolution of gases quenches the plasma with the increase in pressure, which subsequently exfoliates GtO sheets. Other research groups have demonstrated solvothermal reduction of GtO. GtO dispersed in solvents is sealed in an autoclave, and the temperature is raised above the boiling point of the solvent. The high temperature promotes deoxygenation of GtO, thereby reducing it. The solvent used for reduction also plays an important role in determining the dispersibility of GtO in various solvents. The solvents used for reduction of GtO are water, DMF, ethylene glycol, DMSO [40], acetone and sodium hypochlorite solution [41], and alcohols such as methanol, ethanol, isopropanol, and benzyl alcohol [42]. Although there are chemical-reducing agents available for reducing GtO, owing to their toxic potential, their use still remains debatable. Hence, it is highly recommended to follow less toxic, eco-friendly methods to obtain high-quality graphene sheets.

Shear mixer has been demonstrated to exfoliate graphite into individual graphene nanosheets. The combined effect of the rotor and the stator generates shearing, impacting, pressing, turbulence, and cavitation. The mechanical rotation of the rotor creates a shear force that peels off thin GO nanosheets from GtO particles by cleaving the inter-plane bonds. Another force, the impact force, is created owing to the mechanical impact against the rotor, which removes nanosheets from the edges and also induces in-plane fractures [43]. A high-speed shear mixer breaks the van der Waals forces between the adjacent GO layers. A cost-effective hydrodynamic tube shearing was developed by Blomquist et al. [44] to obtain nanographite sheets in an aqueous environment. This method avoids the use of any toxic chemicals, making it eco-friendly.

## Biomedical Applications

Biomedical application of graphene is a new fascinating area that is beyond imagination. The overwhelming properties of GO that support its clinical use are the amphiphilicity, surface functionality, fluorescence quenching ability, and surface-enhanced Raman scattering property. The hydrophobic nature, large surface area, ripples, and grain boundaries on defective sites of graphene are important factors when considering them for biomedical use. The first-ever use of GO as a nanocarrier for drug delivery reported by Sun et al. [45] paved the way to explore the further use of graphene in the biomedical field. Presently, graphene and GO have been known to be used as a carrier for drug delivery, gene therapy, bioimaging, biosensors, and antibacterial composites and as a scaffold for cell culture in tissue engineering.

### Graphene Substrates for Drug Delivery

**Fig. 2:**
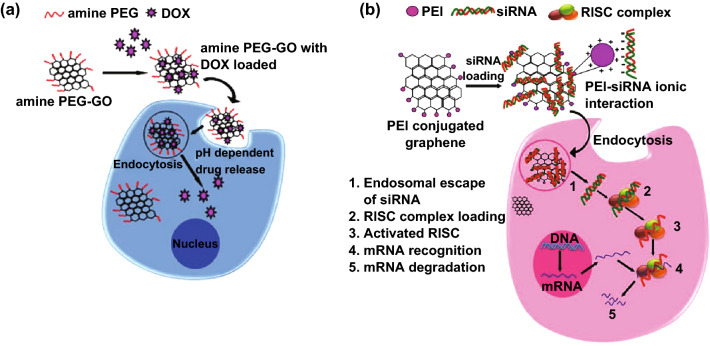
Drug and gene delivery applications. **a** Delivery of doxorubicin using amine-PEG-functionalized GO. **b** siRNA delivery and mRNA degradation using PEI-conjugated GO for gene silencing technology (RISC—RNA-induced silencing complex)

GO with its oxygen-containing functional groups (COOH and OH) has been reported as an effective carrier for drug or gene delivery (Fig. 2a). Despite the presence of functional groups, the high surface area and the basal planar structure with sp^2^ domain afford them high loading capacity, high solubility, and biocompatibility. Multimodal GO with multiple functions can be produced by conjugating polymers, proteins, and biomolecules via simple physisorption or chemical conjugation. GO acts as an efficient nanocarrier for delivering water-insoluble anticancer drugs. The water-insoluble anticancer drug SN38 was successfully loaded onto amine-terminated PEG-grafted GO by noncovalent adsorption and was targeted against cancer cells [46]. The delocalized *π* electron on the graphene surface helps in loading of aromatic anticancer drugs through *π*–*π* stacking or hydrophobic interaction. Selective killing of cancer cells was achieved by loading doxorubicin (DOX) onto antibody-conjugated PEGylated nano-GO (NGO) sheets. The quinone portion of DOX binds to GO via *π*–*π* interactions, whereas hydrogen bond is formed between the amino/hydroxyl groups of DOX and the hydroxyl/carboxyl groups in GO. It was noticed that the drug loading and release kinetics depends on the pH. Maximum drug loading capacity was observed at neutral pH, whereas more than 70% of the drug was released at an acidic pH of 2 [47]. Under acidic pH, the amine group in DOX becomes protonated, resulting in partial dissociation of the hydrogen bond, causing drug release. For improvement in the efficiency of the cellular uptake of DOX, RGO was modified with gold nanoclusters, which strongly inhibit cancer cell growth [48].

GO also improves the solubility and bioavailability of camptothecin (CPT), a quinoline alkaloid that kills cancer cells by inhibiting the DNA enzyme topoisomerase I. Since cancer cells are known to express high amounts of folate receptors, controlled and targeted delivery of multiple drugs is made feasible by conjugating folic acid onto graphene sheets for effective killing of cancer cells. De Sousa et al. [49] utilized folic acid-conjugated GO for the delivery of the chemotherapeutic drug CPT. More than 40% of the loaded drugs were released from the GO-FA surface at 48 h compared to that from the GO surface. Prolonged and sustained drug release over a period of 200 h at physiological pH was observed in the GO surface in their study. This could be due to the stronger interaction between the CPT lactone ring and the aromatic rings of GO. Moreover, this nanocarrier also exhibits pH-dependent drug release with increased release rate at physiological pH compared to acidic pH. Similar extended drug release from a GO nanocarrier coated with folic acid was observed by Saifullah et al. [50]. In their study, protocatechuic acid, a phenolic compound with anticancer property, was conjugated to a GO-PEG nanocarrier coated with folic acid. In contrast to the above study, here, sustained drug release was observed in both physiological and acidic pH. Both studies showed improved anticancer activity for the nanocarrier coated with folic acid compared to the free drug and to the nanocarrier carrying drugs without folic acid. Co-delivery of anticancer drugs such as DOX and CPT was demonstrated using folic acid-conjugated GO complexes [51]. Moreover, pH-sensitive and thermo-responsive drug release from graphene sheets was also demonstrated by several research groups.

Two “off–on” switches of a photoresponsive drug release system made of GO and mesoporous silica nanoparticles (MSNs) were developed [52]. Here, the anticancer drug is loaded inside the MSNs and GO is wrapped around the nanoparticles, which act as a gatekeeper preventing the release of anticancer drugs into nontargeted cells. Furthermore, GO is modified with Cy5.5-labeled AS1411 aptamer that recognizes and binds to the nucleolin of cancer cells. Binding of Cy5.5-labeled AS1411 to GO via hydrophobic interactions quenches its fluorescence. Following endocytosis, the fluorescence is recovered and laser irradiation induces heat that promotes the expansion of GO, thereby releasing drug molecules from the MSNs.

### Graphene Substrates for Gene and Protein Delivery

Gene therapy is a grafting technique to successfully treat various genetic disorders. Successful gene therapy is achieved by developing a vector that protects the DNA from endonuclease and possesses high transfection efficiency. Molecular beacons and aptamers can be delivered inside cells with the help of GO for specific detection of biomolecules. It is well known that cationic polymers such as polyethyleneimine (PEI) induce a proton sponge effect on endocytosis and promote endosomal escape for efficient gene delivery. For the facilitation of gene delivery, GO functionalized with positively charged cationic PEI was transfected with plasmid DNA [53]. The plasmid DNA gets condensed in GO because of the electrostatic interaction resulting from positively charged PEI and negatively charged nucleic acid. This not only forms a stable construct but also improves the transfection efficiency of the vector with decreased cytotoxicity.

Gene silencing in cells can be achieved by delivering siRNA using PEI-GO (Fig. 2b). For example, Zhang et al. [54] developed PEI-GO complexes for the sequential delivery of Bcl-2 targeted siRNA and drug DOX for enhanced therapeutic purposes. The complex exhibited higher cytotoxicity because of the synergistic effect of the drug and siRNA. Similarly, PEG and branched PEI grafted onto NGO sheets were found to have increased transfection efficiency under mild laser irradiation [55]. Heat generated at the irradiated site induces physical disruption of the endosomal membrane, thereby releasing the complexes with increased transfection efficiency. Moreover, PEI-functionalized GO incorporated into GelMA hydrogel was developed for the efficient delivery of the vascular endothelial growth factor (VEGF) gene to promote vasculogenesis and cardiac repair [56]. This study suggests the potential use of a GO-hydrogel system to treat ischemic heart diseases via gene therapy.

Graphene acts as a perfect platform for binding of protein molecules, and it also protects proteins from proteolysis. Hence, graphene-based materials can be used as a nanocarrier for the intracellular delivery of therapeutic proteins. Bone morphogenetic protein (BMP) is a well-studied osteoinductive protein for inducing bone regeneration. Local delivery of BMP to induce osteogenesis for bone regeneration is highly appreciable in clinical treatment. GO-coated Ti implant was developed as a vehicle for the delivery of BMP at the site of interest [57]. Sustained release of BMP at the implanted site was achieved through GO coating. Co-delivery of BMP and substance P using a GO-Ti implant recruited mesenchymal stem cells (MSCs) toward the implanted site and promoted bone formation in a mouse calvarial defect model. Emadi et al. [58] developed GO modified with chitosan (GO-CS) as a protein therapeutic nanocarrier for the successful delivery of protein during intravascular and oral administration. BSA and collagenase are loaded onto chitosan-functionalized GO. In this approach, chitosan-modified GO protects BSA from proteolytic cleavage and also retains the enzymatic activity of collagenase. The protective effect of GO-CS on BSA from trypsin digestion is explained as being due to the steric hindrance of GO as well as due to the reducing effect of BSA. BSA removes oxygen-containing functional groups from GO; the reduced GO thus formed aggregates and wraps around trypsin, thereby preventing their interaction with BSA. Therefore, this study [58] promises the potential use of a GO-based nanocarrier for enhanced protein delivery that reduces the overall therapy cost by improving the therapeutic efficiency and also by reducing the frequency of repeated administration.

### Graphene Substrates for Photothermal Therapy and Photodynamic Therapy

Photothermal therapy (PTT) is recently considered as a minimally invasive and highly efficient method for cancer treatment. PTT involves the conversion of light energy into heat energy by certain light-absorbing agents under irradiation. When the excited molecules come to a ground state, they release energy in the form of heat that causes thermal ablation of cancer. Nanomaterials with NIR absorbance are highly appreciable for PTT as they avoid interference from biological tissues. Compared to that of the commonly employed PPT agents such as gold nanoparticles, the lower power density (2 W cm^−2^) of pristine GO provides effective PTT efficiency in vivo [59]. In addition, the high surface area available in graphene offers efficient drug loading capacity and conjugation of ligand molecules to achieve targeted and enhanced therapeutic potential. Moreover, biofunctionalization of graphene with FBS, PEG, and dextran improves its biocompatibility [60], enhances it photothermal efficiency, and increases the blood circulation time and bioavailability inside the body. In addition, certain fluorescent dyes or molecules such as indocyanine green (ICG), phthalocyanine, and quantum dots can be conjugated onto graphene to achieve imaging-guided therapy.

**Fig. 3:**
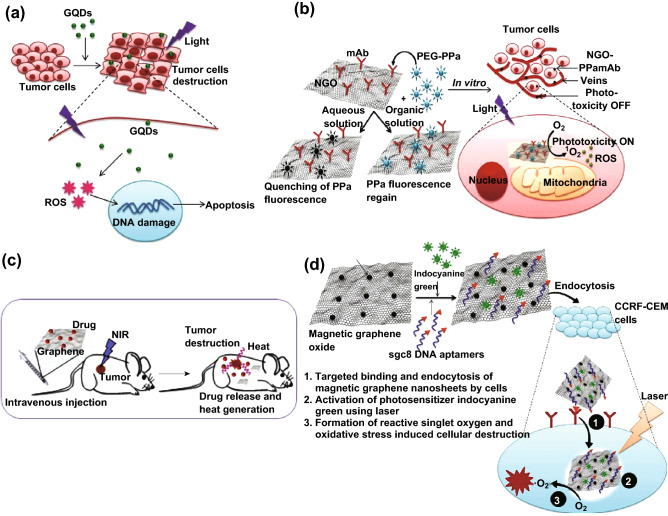
Anticancer therapy. **a** Photothermal destruction of tumor cells following GQD exposure and irradiation. **b** Development of the phototoxicity on/off system for subcellular targeting and selective destruction of tumor cells. **c** Combined chemo- and photothermal destruction of tumor cells using drug-loaded graphene nanosheets under NIR irradiation. **d** Photodynamic therapy using magnetic GO nanoconjugates functionalized with aptamer and an ICG photosensitizer to achieve targeted cancer cell destruction

GO shows higher absorbance in the NIR region, a property that is utilized for photothermal destruction of tumor cells (hyperthermia). PEGylated nanographene sheets showed high tumor uptake efficiency when injected into a mouse tumor model [61]. It also exhibited high retention time owing to enhanced permeability and tumor destruction (Fig. 3a, c). Efficient tumor ablation following intravenous administration (20 mg kg^−1^) and low-power NIR irradiation (808 laser, 2 W cm^−2^) was noticed in a 4T1 tumor mice model. Complete destruction of the tumor in 1 day was achieved, with no tumor regrowth observed for another 40 days. GO decorated with iron oxide and gold nanoparticles was fabricated to obtain a multifunctional nanocomposite with strong superparamagnetism and enhanced NIR absorbance. The biocompatibility of the nanocomposite was further increased by surface functionalization with PEG. This multifunctional nanocomposite showed remarkable photothermal ablation of cancer with decreased toxicity. Magnetic resonance (MR) and X-ray dual-modal imaging were made possible owing to the presence of iron oxide and gold nanoparticles [62]. It was also reported that irradiation of GO nanoparticles under femtosecond laser beam induces the formation of microbubbles [63]. Irradiation with laser increases the temperature and simultaneously reduces GO nanoparticles. The removal of oxygen groups from GO is accompanied by the release of CO_2_ and H_2_O. The instant formation and collapse of microbubbles damage the cancer cells.

Combined chemo- and photothermal therapy is an effective way to treat cancer compared to monotherapy. The high drug loading capacity and NIR absorbance property of graphene can be utilized for the synergistic treatment. To achieve this, the authors in [64] developed a poly-dopamine-functionalized reduced graphene oxide (PDA-RGO) nanocomposite that exhibited excellent biocompatibility, effective photothermal efficacy, high drug loading capacity, and sustained drug release. In another study, BSA-functionalized RGO was loaded with DOX and exposed to U87MG cells [65]. Absorption of NIR by RGO generates heat that destroys the binding between DOX and BSA; as a result, twice the amount of DOX will be released from the nanosheets.

Photodynamic therapy (PDT) is a novel treatment for cancer as it combines photosensitizer-mediated targeted killing. Upon light activation, the photosensitizer molecule gets excited and induces the formation of reactive oxygen species (ROS) that cause irreversible damage to cancer cells. Chlorine 6, a photosensitizer that is loaded onto folic acid-conjugated GO via hydrophobic and *π*–*π* stacking, showed effective killing of MGC803 cancer cells under irradiation [66]. Porphyrin, a well-known photosensitizer, was also functionalized onto GO nanosheets through *π*–*π* interaction. This complex can be utilized to treat tumors under hypoxic conditions as well as deep solid tumors such as the glioblastoma multiform tumor [67]. PEG-GO carrying 2-(1-hexyloxyethyl)-2-devinyl pyropheophorbide-alpha is a photosensitizer currently under investigation in phase I and phase II clinical trials for the treatment of various types of cancer. This complex has been found to have high loading capacity and improved PDT [68]. Ultrasound is also used to activate the sensitizer instead of light, which has the advantage of deep tissue penetration compared to PDT. Simultaneous imaging and therapy are achieved by tagging graphene sheets with fluorescent dyes (phthalocyanine and folic acid), inorganic nanoparticles (iron oxide and gold nanorods), and quantum dots [69]. Aptamer-conjugated magnetic GO nanosheets loaded with the photosensitizer ICG has been developed for targeted photothermal and photodynamic therapy [70]. ICG, a near-infrared dye, was incorporated onto the surface of magnetic GO nanosheets via *π*–*π* stacking. This was further modified with aptamer sgc8, which specifically binds to the protein expressed by CCRF-CEM cancer cells. These nanoconjugates are effectively endocytosed by the cells, and upon laser irradiation, they induce the formation of heat and singlet oxygen (Fig. 3d), resulting in dual photothermal and photodynamic therapy. This system kills almost 82% of cancer cells (at 100 ppm of nanoconjugates) when irradiated with a laser for 5 min. Recently, Dos Santos et al. [71] developed an NGO-methylene blue conjugated system for the effective killing of breast cancer cells. In their study, they conjugated an inexpensive FDA-approved methylene blue dye to NGO stabilized with Pluronic F127. Methylene blue, a hydrophilic dye that absorbs light at a wide wavelength range and produces singlet oxygen species upon irradiation, has been approved by the FDA for the treatment of methemoglobinemia. Moreover, in their study, the combined PTT/PDT efficiency of NGO-methylene blue complex was evaluated in mice carrying 4T1-Luc cells as a model for human later-stage breast cancer. At physiological pH (pH 7.4), methylene blue interacts with NGO through electrostatic interactions. It was noticed that the release of methylene blue was higher at acidic pH (pH 5.0) than at a pH of 7.4. However, under acidic condition, the carboxylate group of NGO becomes protonated, which, in turn, weakens its interaction with methylene blue, resulting in faster release of the dye molecule. As mentioned before, absorption of NIR radiation by NGO increases the solution temperature to a maximum of 60 °C, which is sufficient for hyperthermia treatment. Released methylene blue inside the tumor cells produces a significant amount of ROS upon irradiation. These findings promise the potential use of this system for complete tumor ablation and metastasis prevention. Several research groups have designed hybrid materials comprising photosensitizer-linked graphene/GO (GO-fullerene C60) [72], RGO-Ru-PEG [73], folic acid-GO-manganese dioxide [74], GO-enwrapped SiO_2_/TiO_2_ hollow nanoparticles loaded with protoporphyrin IX [75], and NGO-UCNP-Ce6 [76] for combined PTT and PDT in a single platform.

Effective delivery of photosensitizers for PDT along with enhanced fluorescence imaging was achieved by Yan et al. [77]. In their study, PEGylated GO was loaded with a novel photo-theranostic agent based on sinoporphyrin sodium (DVDMS). It was found that GO augments the accumulation of the photosensitizer in the tumor, demonstrating 100% tumor elimination without visible toxicity. The intramolecular charge transfer between the porphyrin rings of DVDMS enhances its fluorescent property, acting as a dynamic molecule for optical imaging-guided PDT.

Similarly, Ce6 (aromatic photosensitizer) was effectively conjugated onto the surface of polyvinylpyrrolidone (PVP)-functionalized RGO (nanocarbon) for effective PDT [78]. Coating with PVP improves the biocompatibility and aqueous stability and offers a site for RGD peptide linkage. This nanocarbon ensures increased accumulation in target cells with improved efficacy. Likewise, folic acid-conjugated GO carrying Ce6 showed a significant increase in tumor accumulation and remarkable photodynamic efficacy under irradiation [66]. This conjugate system was found to enter into the cells via endocytosis and accumulated in the lysosomes. Owing to the acidic environment in the lysosomes, Ce6 is released into the cytosol and, under irradiation, induces efficient photodynamic activity. Targeted PDT can be achieved by functionalization of graphene sheets with specific ligands that bind to tumor cells expressing specific receptors on their surfaces, thereby potentially avoiding toxicity to the neighboring cells. Moreover, subcellular localization of the photosensitizer-loaded graphene in specific organelles was also demonstrated by Wei et al. [79]. Nanographene oxide conjugated with monoclonal antibodies (mAb) of integrin receptors ανβ3 was loaded with PEG-PPa (pyropheophorbide-a). PEG-PPa binds to GO via *π*–*π* stacking. Upon irradiation, the fluorescence of PPa is quenched owing to the energy transfer from PPa to GO through the fluorescence resonance energy transfer (FRET) mechanism. PPa-NGO-mAb enters into the cell and accumulates in the mitochondria. While entering the mitochondria, PPa-NGO-mAb comes into contact with the lipid mitochondrial membrane where *π*–*π* interaction is broken, resulting in fluorescent regain of PPa. This phototoxicity on/off system based on a graphene material is a novel carrier for subcellular targeting and attacking of αvβ3-expressing tumor cells via the production of singlet oxygen (Fig. 3b). Table 1 summarizes the different graphene-based materials developed for cancer therapy.

**Table 1:** Different graphene materials for cancer treatment (in vitro and in vivo studies)

Graphene materials	Drugs	Cell lines/animal models	Refs.
Glucose-RGO	–	LNCaP prostate cancer cells	[31]
GO-azoaromatic crosslinkers- (PVA)	Curcumin	Colon cancer	[80]
GO-PEG	Paclitaxel	A549, MCF-7 cells	[81]
Pluronic F127/graphene nanosheet	Doxorubicin	MCF-7 cells	[82]
Chitosan-GO	Camptothecin (CPT)	HepG2 and HeLa cells	[83]
Dextran-modified GO	Curcumin	4T1 mammary carcinoma cell line	[84]
AuNPs–GO	Doxorubicin	HepG2 cells	[85]
GQDs- FA	Doxorubicin	HeLa cells, A549, and HEK293A	[86]
GQDs- Biotin	Doxorubicin	A549 cells	[87]
GQDs- herceptin	Doxorubicin	Breast cancer cells	[88]
AuNPs/RGO composites	Mitoxantrone	MCF-7 breast cancer cells	[89]
RGO coated Cu_2−x_Se nanoparticles	Doxorubicin	HEp-2 and A549 cells	[90]
Dextran-FA-RGO	Doxorubicin	HeLa cells	[91]
RGO-LHT7	Doxorubicin	Human KB carcinoma cells (in vitro and in vivo)	[92]
PEG-GO	Doxorubicin	SCC7 cells (in vitro) and in vivo	[93]
GO–Silver nanocomposite	Salinomycin	Human ovarian cancer stem cells	[94]

*RGO* Reduced graphene oxide, *GO* graphene oxide, *PVA* poly (vinyl alcohol), *PEG* polyethylene glycol, *AuNPs* Gold nanoparticles, *GQDs* graphene quantum dots, *FA* folic acid, *LTH7* low molecular weight heparin

### Graphene Substrates as Biosensors

**Fig. 4:**
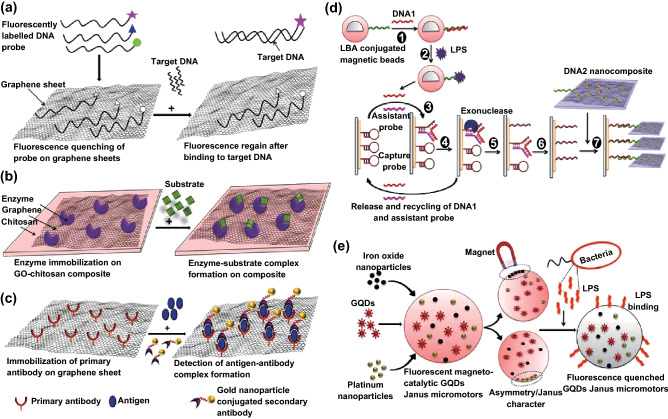
Graphene-based biosensors. **a** FRET-based graphene sensors for the detection of target DNA using fluorescent-labeled ssDNA. **b** Graphene biosensors for the immobilization of enzymes to monitor enzyme activity. **c** Graphene immunosensor for the detection of an antigen–antibody complex for early disease diagnosis. **d** Graphene nanohybrid electrochemical aptasensor for endotoxin detection. Step 1: Complementary DNA 1 is hybridized to LBA. Step 2: LPS binds to LBA, promoting the release of DNA 1. Step 3: Together with the help of an associated probe, DNA 1 binds to a capture probe and forms a Y-shaped structure. Step 4: Exonuclease nicking cleaves the capture probe and releases both DNA 1 and the assistant probe. Step 5: DNA 1 and the assistant probe are rehybridized to the new capture probe, and the cycle continues. Step 6: More numbers of cleaved capture probes are formed. Step 7: Capture probes are hybridized to DNA 2 nanocomposites, resulting in the production of electrochemical signals. **e** Structure of the magnetocatalytic GQD-based Janus micromotors and fluorescence quenching upon binding of LPS

Graphene has attractive application in the development of electrochemical sensors and biosensors owing to its excellent electrical conductivity, large surface area, and high electron transfer potential. Graphene-based field effect transistor biosensors have been developed to detect biomolecules such as nucleic acids, proteins, and growth factors, which monitor the changes in an electrical signal. Developing graphene-based biosensors relies on the fluorescent quenching property of graphene. The detection of nucleic acid is done by tagging GO with fluorescent-labeled ssDNA. GO quenches the fluorescence of ssDNA. This ssDNA forms a double helical structure when it comes into contact with the target complementary sequence. The formation of a double helix displaces GO from an ssDNA strand, resulting in fluorescence recovery (Fig. 4a).

FRET biosensors, FET biosensors, and biosensors for DNA detection [95] have been developed with graphene-based materials. For example, nitrogen-doped graphene FET biosensors for detecting VEGFs [96] and biosensors for detecting catecholamines (dopamine, epinephrine, and norepinephrine) were developed. Detection of important factors of human metabolism such as ascorbic acid and uric acid remains a challenge in diagnostic and pathological research. Several graphene-based sensors were developed for the simultaneous detection of these factors [97]. CVD graphene FET biosensors were developed for detecting electrical signals from electrogenic cells (cardiomyocytes) [98]. Proteins, hormones, adenosine triphosphate (ATP), fungi toxins, and harmful metal ions can be detected using graphene-based biosensors. GO biosensors for measuring the activity of several enzymes such as caspase-3, trypsin, thrombin, and metalloproteinase and the activity of DNA helicase [99] were also developed (Fig. 4b).

Graphene-based biosensors for the detection of pathogens have also been developed. Huang et al. demonstrated a nanoelectronic biosensor for the detection of *Escherichia coli* (*E. coli*) [100]. CVD-grown graphene film was functionalized with anti *E. coli* antibodies and was passivated using Tween 20. This graphene biosensor detects *E. coli* with high specificity and selectivity. The conductance of the graphene sensor increases with the increase in the number of *E. coli* attached on the graphene sheets. In addition, graphene is also used for the sensitive detection of biomarkers for early detection of life-threatening diseases (Fig. 4c). In the case of cancer biomarker detection, the primary antibody against prostate-specific antigen is immobilized on graphene sheets. In the presence of antigen, an immunocomplex is formed between GO-Ab 2 and magnetic bead-Ab 1. Addition of hydroquinone and H_2_O_2_ solution to the immunocomplex results in color development. The extent of color changes correlates with the amount of antigen present [101]. Use of graphene for the fabrication of immunosensors in disease diagnosis offers several advantages: high surface-to-volume ratio of graphene sheets, which enables high immobilization of primary antibodies; good electrical conductivity, which promotes good electron transfer; low detection limit; and enhanced sensitivity and reproducibility.

Graphene-based biosensors have also been developed for the detection of lipopolysaccharides (LPSs). The most commonly employed enzymatic assay for LPS detection is the limulus amebocyte lysate (LAL) assay. However, the LAL assay has certain pitfalls: it is highly susceptible to changes in temperature and pH and requires monotonous sample preparation and controlled experimental conditions. Several efforts have been taken to develop alternate methods for the detection of endotoxin using synthetic sensors (FRET sensors, aptamer sensors, and cell-based biosensors). However, these sensors are expensive, are not robust, and are less sensitive to the detection of endotoxin at the picomolar regime. A GO-based fluorescence turn-on biosensor was developed for the detection of endotoxins [102]. Tetramethylrhodamine dye-labeled LPS-binding peptide was physically adsorbed on the surface of GO via electrostatic interaction or *π*–*π* stacking. The adsorption of the peptide to GO quenches the fluorescence emission from the dye molecule. Competitive binding of LPS induces fluorescence recovery, releasing dye-labeled peptide from the GO sheets. This method is a rapid, selective, and sensitive method for the detection of LPS/endotoxin in aqueous solution at room temperature. The detection limit of the sensor was found to be 130 pM.

Bai et al. [103] demonstrated an electrochemical aptasensor for the ultrasensitive detection of endotoxins. This technique combines the three-way DNA hybridization process and nanotechnology-based amplification. In short, LPS-binding aptamer (LBA)-conjugated Au@Fe_3_O_4_ magnetic beads were hybridized to a complementary DNA 1 probe. Incubation of these magnetic beads in LPS solution promotes the binding of LPS to LBA aptamer, concomitantly releasing DNA 1. The released DNA 1 together with the help of an assistant probe binds to the capture probe and unwinds its hair pin structure, forming a Y-shaped junction structure. This Y-shaped capture probe is then cleaved by exonucleases, releasing both the DNA 1 and the assistant probe. The released probes are now available for binding to new capture probes and the cycle continues. The continuous action of rehybridization, nicking, and release creates a large number of cleaved capture probes. The cleaved capture probes bind with a DNA 2 nanocomposite. DNA 2 nanocomposite is made of DNA 2-labeled AuNPs-Tb-Gra. The large surface area of graphene increases the immobilization of toluidine blue (Tb) and enhances the electrochemical signal. Both graphene and gold nanoparticles (AuNPs) amplify the signal, providing the detection of LPS at the femtogram level (Fig. 4d).

Recently, magnetocatalytic graphene quantum dot (GQD)-based Janus micromotors were synthesized for the detection of endotoxin released from *E. coli* bacteria (LPS 0111:B4) [104]. These micromotors are composed of phenylboronic acid (PABA)-encapsulated GQDs together with platinum and iron oxide nanoparticles on one side. The presence of platinum and iron oxide nanoparticles enables the autonomous propulsion of micromotors in the presence of hydrogen peroxide or magnetic field without the addition of chemical fuels. The PABA of GQDs specifically recognizes LPS in contaminated urine and serum samples, and the interaction between GQDs and endotoxin results in fluorescence quenching of GQDs (Fig. 4e). The extent of fluorescence quenching was directly proportional to the concentration of LPS. An obvious decrease in fluorescence intensity was noticed even at 0.01 M concentration of LPS, with 100% fluorescence quenching observed at 1 M concentration.

### Bioimaging Application of Graphene Derivatives

**Fig. 5:**
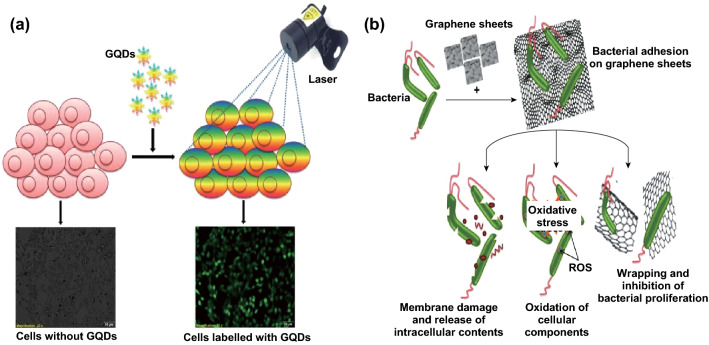
Bioimaging application. **a** Graphene quantum dots for in vitro cellular imaging. **b** Mechanism of toxicity induced by graphene in bacterial cells

Since GO possesses strong absorbance and fluorescence property in the NIR region, it is used as an imaging tool. Fluorescence can be induced in GO by manipulating the synthesis conditions such as the pH, rate of reduction, and size. The presence of functional groups on the sides of the planar graphene can be conjugated with fluorescent dyes for bioimaging. Simultaneous imaging and drug delivery using GO were recently reported by Cheng et al. [105]. Under mild thermal annealing, GO was shown to emit blue fluorescence owing to the formation of *sp*^2^ and oxidized domain. Annealing induces phase transformation in GO that promotes oxygen diffusion, resulting in the formation of nanosized (1-1.5 nm) graphitic domains responsible for the blue photoluminescence. This procedure permits oxygen diffusion only and does not remove oxygen from the graphitic structure; hence, subsequent conjugation of drug molecules is also possible. Jin et al. [106] stated that GO in nanoform shows photoluminescence properties, which arise from the quantum confinement effect utilized for bioimaging purposes. GO with a size less of than 10 nm described as a GQD is prepared from GO. GQDs are also prepared from pre-oxidized graphene sheets by hydrothermal cutting and oxygen plasma treatment. Compared with conventional fluorescent probes and other QDs, GQDs possess high stability, excellent biocompatibility, good solubility, and low cytotoxicity that can be utilized for both in vitro and in vivo imaging (Fig. 5a). GQDs also exhibit up-conversion fluorescence (excitation at NIR region) that reduces interference from autofluorescence [107].

GO protects DNA from hydrolysis by DNAse I owing to the steric hindrance preventing the binding of DNAse I. Hence, graphene can be used for the delivery of aptamer probes for in vivo imaging of biomolecules. In a study [108], GO nanosheets were conjugated using aptamer-carboxyfluorescein (FAM) for imaging ATP and GTP in live cells. Binding of aptamer to GO quenches the fluorescence. After the cellular uptake, the aptamer detects ATP and the complex interaction changes the structure of aptamer recovering the fluorescence. Graphene nanosheets were linked with Cy7 for in vivo fluorescent imaging [61]. The study showed increased tumor uptake of nanosheets in xenograft 4T1 murine breast cancer tumor, KB human epidermoid carcinoma tumor, and U87MG human glioblastoma tumor mouse models. Next to fluorescent imaging, MRI is one of the most widely used imaging methods for clinical purposes. Super paramagnetic Fe_3_O_4_ nanoparticles were immobilized on GO nanosheets and were used as a contrast agent in MRI, which not only exhibited enhanced MRI signal but also were biocompatible [109].

### Graphene Derivatives as an Antimicrobial Agent

Both GO and RGO are known to possess antibacterial activity against a wide range of bacteria. It was found that Gram-negative bacteria such as *E. coli* are less sensitive to graphene than Gram-positive bacteria such as *Staphylococcus aureus* [110]. The presence of the outer membrane on Gram-negative bacteria protects them from cellular damage. Graphene exhibits antibacterial activity by directly interacting with the cell membrane. Liu et al. [111] proposed a three-step mechanism for the antibacterial action of graphene: (1) Bacteria attach to the surface of graphene sheets; (2) membrane is damaged, resulting in leakage of the intracellular contents; and (3) membrane lipids and proteins are oxidized (Fig. 5b).

RGO nanowalls were found to be more toxic toward bacteria than GO because of better charge transfer with the bacterial cells and of the presence of sharp edges of RGO [112]. The sharp edges induce membrane perturbation, leading to leakage of the intracellular contents. Graphene also acts as a good electron acceptor and prevents electron transfer in the electron transport chain (ETC), resulting in depletion of ATP and, eventually, in cell death. For Gram-negative bacteria, the minimum inhibitory concentration (MIC) was found to be 1 µg mL^−1^, whereas for Gram-positive bacteria, MIC was found to be 4–8 µg mL^−1^ [113]. Graphene induces the formation of ROS inside the cells, which damage cellular components such as DNA, lipid, and protein. Lipid peroxides are formed as a result of fatty acid oxidation, which disintegrates cell membrane, eventually leading to cell death. It was also proposed that the physical contact between the bacterial cell membrane and semimetal graphene facilitates the charging of the electron from membrane to graphene. The interruption of electron transfer in the respiratory chain results in depletion of intracellular ATP. Graphene extracts electrons until the bacteria lose their viability. Several graphene composites comprising polymers (poly-l-lysine, chitosan, lactoferrin, and polyvinyl-*N*-carbazole) have also been developed to provide antibacterial surface for biomedical applications. Recently, Zarafu et al. [114] functionalized GO with amine-containing organic compounds and investigated the antimicrobial and antibiofilm activity of Gram-negative (*Escherichia coli* and *Pseudomonas aeruginosa*) and Gram-positive (*S. aureus*) bacteria. These functionalized GO hybrids exhibited improved inhibitory activity against bacteria compared to amines alone.

The antibacterial activity of GO photolyzed under simulated sunlight was studied recently by Hou et al. [80]. To induce phototransformation of GO, they irradiated the samples after the preparation under simulated sunlight for different time periods. Two approaches for phototransformation were followed: direct (photolyzed under sunlight) and indirect (GO containing H_2_O_2_) photolysis. Direct photolysis samples were more effective in inhibiting the growth of the bacteria *E. coli* K12 than the indirect photolyzed samples. These differences could be attributed to the size of GO after photolysis. Indirect photolysis that involves addition of H_2_O_2_ resulted in the formation of smaller GO sheets than those formed using direct photolysis. The larger GO from direct photolysis can effectively interact, wrap the cells, and induce membrane deformation. In addition, it also possesses greater oxidation capacity toward GSH, depleting the cellular antioxidant level. Another study demonstrated the use of GO as a reservoir for loading of antimicrobial peptide (G(IIKK)_4_I-NH_2_) and for its sustained release [115]. The positively charged G(IIKK)_4_I-NH_2_ enhances its binding on the negative charge surface of GO. Upon binding, G(IIKK)_4_I-NH_2_ monomers undergo structural transition to form an α-helix secondary structure. Layer-by-layer assembly of a GO-G(IIKK)_4_I-NH_2_ nanocomposite showed similar MIC values to those obtained from free G(IIKK)_4_I-NH_2_. Sustained release of antimicrobial peptide from this nanocomposite demonstrates an effective approach for surface coating of devices to achieve long-term antibacterial activity.

In contrast to the above findings, enhanced bacterial growth was observed on the GO surface [116]. The bacterial growth was found to be three times higher on the GO modified surface, with a greater number of bacteria on the surface with high particle density. It is suggested that the oxygen groups of GO confer enough wettability for bacterial adhesion and proliferation. Despite the contradictory findings, the antimicrobial property of graphene-based materials can be utilized for surface coatings of nanocomposites, for wound dressings [117], on medical device surfaces, and as smart antibiotics [118], after a thorough investigation.

### Graphene Substrates for Tissue Engineering

**Fig. 6:**
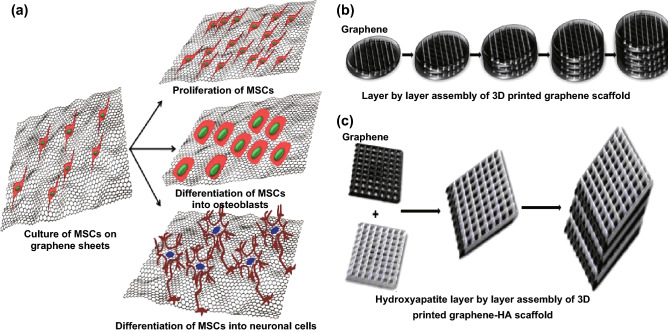
Graphene as a scaffold for tissue engineering. **a** The figure explains that graphene enhances the viability and proliferation of mesenchymal stem cells and also induces their differentiation toward osteogenesis and neuronal cells without the addition of any differentiation factors. **b** Layer-by-layer assembly of graphene sheets printed using a 3D printer. **c** Three-dimensional printing and layer-by-layer assembly of a graphene-HA scaffold

Successful tissue engineering depends on the biocompatible substratum that offers cells to attach, grow, and proliferate. Stem cells are one of the most promising candidates for tissue regeneration because of their differentiation into cells of specific lineage. Recently, graphene has been put on the spotlight as a reliable scaffold for the attachment and proliferation of stem cells, especially MSCs and neuronal cells. Several cell lines were cultured on the surface of graphene-coated substratum, e.g., osteoblasts [119], NIH-3T3 cells [120], MCF-7 cells [121], and MSCs. MSCs grown on graphene surface/3D graphene foam attach and form a spindle shape with high proliferation and differentiation potential toward osteogenic lineages (Fig. 6a) without the addition of any external biochemical cues. The concentration of graphene should be overlooked while developing the scaffold for cell culture since it is a critical factor that determines cell viability. It is believed that a lower concentration of GO seems to promote cell adhesion and is biodegradable, whereas a higher concentration decreases cell attachment and induces oxidative stress-mediated cytotoxicity. It was explained that graphene acts as a platform for the binding of differentiation-inducing factors. Graphene suppresses adipogenic differentiation, which is due to the fact that insulin, a key inducer in adipogenesis, is denatured upon binding to graphene (*π*–*π* interaction), whereas GO does not affect adipogenesis owing to electrostatic binding of insulin. However, graphene enhances osteogenesis by pre-concentrating osteogenic factors [122].

Another important property of graphene is its electrical conductivity. This property enables graphene to modulate neural stem cell activity. The findings suggested that graphene differentiates human neural stem cells into neurons (Fig. 6a). Neural stem cells are readily and firmly attached on graphene, which promotes their differentiation into neurons rather than into glial cells. Graphene promotes the sprouting of neurites and also increases the number of neurites in mouse hippocampal neural cells along with increased GAP-43 protein expression [123]. The number of neurites and their average length increase in pristine graphene culture compared to those in tissue culture polystyrene substrate. Various graphene-based composites have been developed and tested for their biocompatibility, e.g., graphene and PCL (poly-ε-caprolactone) [124], graphene and chitosan [125], graphene-based porous hydrogel scaffolds [126], and GO-polypropylene carbonate nanofoams [127]. Electron-spun PCL nanofibers coated with GO have been developed for the differentiation of neural stem cells toward oligodendrocytes with high expression of myelin basic protein, Olig2, O4, and GalC. Furthermore, it was described that the scaffold promotes oligodendrocyte differentiation by regulating the downstream signaling pathway of an integrin receptor and associated cytoskeletal remodeling [128]. Engineered cardiac patches have been developed to replace a portion of damaged cardiac tissue. To trigger the regeneration of the myocardium, a successful cardiac patch should provide mechanical strength and supply cells with growth factors to improve cardiac function. Several studies have incorporated graphene-based materials into polymeric scaffolds that are used as cardiac patches to improve their mechanical properties and electrical conductivity. GO-gold nanosheet-incorporated chitosan scaffold [129], RGO-GelMA hybrid hydrogels [130], RGO-nanofibrous silk fibroin matrices [131], GO-incorporated collagen scaffold [132], and graphene–polycaprolactone scaffold [133] have been fabricated for cardiovascular applications. Among the graphene-based materials, RGO has been found to be a more suitable material for cardiac tissue engineering as it provides better electrical conductivity, mechanical properties, and biocompatibility.

The unique mechanical and physical properties of graphene and its ability to induce differentiation of stem cells unlock opportunities in dental applications. High mechanical strength, durability, and biocompatibility are the prerequisite for any dental materials. Fabrication of graphene-based materials into dental composites will increase the mechanical properties such as the compressive strength and the compressive modulus [134]. GO-induced upregulation of odontogenic genes such as dentin matrix acidic phosphoprotein 1 and dentin sialophosphoprotein in stem cells isolated from dental pulps was recently described by Rosa et al. [135]. Dental pulp stem cells were also able to attach and proliferate on the rough GO substrate.

Induced pluripotent stem cells (iPSCs) are another source of cells that have major potential in tissue engineering and regenerative medicine. Both graphene and GO were shown to induce spontaneous differentiation of iPSCs into cells of ectodermal and mesodermal lineages [136]. However, GO and graphene support distinct pathways of cellular differentiation. iPSCs attach rapidly and proliferate at a faster rate in GO. Graphene prevents cellular differentiation toward endodermal lineages, whereas GO supports endodermal differentiation. This could be due to the difference in surface groups that activate different receptors of iPSCs. This study [136] suggests that graphene materials can be used as a substrate for iPSC culture and expansion, which eliminate the need for feeder layer cells. Moreover, it supports that graphene scaffold can be used for cell replacement therapy in acute liver failure or type I diabetes since graphene augments differentiation toward hepatocytes and insulin-producing *β* cells. Altogether, the above findings show the promising use of graphene at a safe dose for stem cell therapy and regenerative medicine.

### Application of Graphene and Its Derivatives in 3D Printing

Three-dimensional (3D) printing is a revolutionary technology, having promising application in tissue and organ engineering. Fabrication of 2D graphene into a 3D structure is made possible with the help of 3D printing. Three-dimensional printing is an efficient technology for enabling the direct production of 3D bulk objects. In this technology, polymer, ceramics, or metals can be heated and deposited layer by layer under computer control to build 3D monoliths that are designed using software associated with the printer. The development of 2D and 3D printing graphene-based ink has helped scientists and engineers develop modern devices, sensors, and constructs for tissue engineering. Three-dimensional graphene printed materials possess high electrical conductivity (> 870 S m^−1^) and high tensile strength (< 1 MPa), are mechanically resilient, possess the ability to withstand strain (> 80%), and are highly bioactive [137]. These properties will greatly expand the versatility of graphene materials for emerging biomedical applications. The study [137] found that graphene-enhanced nanocomposite materials greatly improve the traditional materials used in 3D printing, such as plastics.

A multilayer graphene structure can be developed by depositing 3D printable graphene ink using a predefined patterned object (Fig. 6b). Moreover, multiple sheets of graphene can be rolled, folded, or cut into different shapes. Three-dimensional graphene ink is composed of graphene, solvent, and an elastomeric polymer binder. These 3D graphene inks are user friendly, print rapidly, and exhibit functional material properties. A liquid suspension of mixture (GO, polymer, and solvents) is extruded from the nozzle that rapidly solidifies into a defined structure. Evaporation of the solvent will drive the solidification of the structure.

A solvent-based graphene ink was developed to print 3D graphene structures comprising high graphene content and a polymer polylactide-co-glycolide (PLG) [138]. The 3D graphene (3DG) structure provides an electrically conductive surface and a flexible structure for tissue engineering applications. Apart from this, PLG encompassed in 3DG is a biocompatible and biodegradable polymer. The porosity of 3DG can be tailored to obtain a desirable cell response. Furthermore, human MSCs cultured on the 3DG remain viable and the cells proliferate to coat individual struts and span the interstrut gaps. Cellular morphology studies revealed the differentiation of human MSCs toward a neuronal lineage. Moreover, gene expression analysis studies showed an upregulation of glial and neurogenic relevant genes such as glial fibrillary acidic protein, neuron-specific class III β-tubulin (Tuj1), nestin, and microtubule-associated protein 2 over the course of 2 weeks. Cells formed a wire-like structure as seen in neurons with axon-like extensions. It was considered that the high graphene content in 3DG (60 vol% graphene) induces MSC differentiation toward neuronal lineages without any additional factors.

In vivo studies in mice showed that a subcutaneously implanted 3DG scaffold had no severe immune response or fibrous capsule formation. Pores of the scaffold were covered with extracellular matrix structure collagen. Vascularization and tissue integration were noticed after 30 days of implantation. Macrophages appeared to surround the implanted site, which probably indicates the removal of graphene sheets. Absence of pathological lesions in organs (kidney, spleen, and liver) indicates that the graphene sheets either were cleared away from the system or remained in close proximity to the implanted site.

Similarly, GO was incorporated into a nanocomposite based on polyurethane/poly lactic acid, which increases the mechanical strength and thermal property of 3D nanocomposites. It was found that the mechanical strength depends on the printing orientation. It was also revealed that the nanocomposite provides good biocompatibility with NIH3T3 cells, promising their potential use as a biomaterial scaffold in tissue engineering [120]. Multi-compositional and multifunctional structures can be readily made with the help of 3D printing for complex tissue engineering applications. Three-dimensional printed biomaterials composed of hydroxyapatite (HA) microspheres and graphene nanoflakes were co-printed [139]. These hybrid materials are electrically conductive and flexible, and also exhibit the characteristics of both materials (Fig. 6b). This 3D construct supports cell viability and proliferation of mesenchymal stem cells, and it upregulates osteogenic and neurogenic gene expression significantly. Hence, with the help of 3D printing, an array of biomaterials can be tuned to obtain desirable properties and multiple functionalities for complex tissue engineering applications as well as to fabricate surgery-friendly constructs. Table 2 shows a few examples of graphene materials with potential biomedical applications.

**Table 2:** Biomedical applications of the graphene family materials

Graphene materials	Drug/gene/antibody	Cells/animals	Applications	Refs.
PEG-NGO	Anti-CD20	Raji B cell lymphoma	Anticancer treatment	[45]
PEG-NGO	SN38	Human colon cancer cell line—HCT-116	Anticancer treatment	[46]
Gold nanoclusters-RGO	Doxorubicin	HepG2 cells	Drug delivery, cellular imaging	[48]
FA-GO-PEG	Protocatechuic acid	HEP-G2 cells; HT-29 cells	Anticancer treatment	[49]
FA-NGO	Doxorubicin Camptothecin	MCF-7 cells	Targeted drug delivery	[51]
Aptamer-GO-wrapped mesoporous silica nanoparticles	Doxorubicin	MCF-7 cells	Photoresponsive drug release; Anticancer treatment	[52]
PEI-GO	Enhanced green fluorescence protein, Bcl-2-targeted siRNA.	HeLa cells, HepG2 cells, HEP-2 cells	Gene delivery, siRNA delivery	[53, 54]
PEI-GO-GelMA hydrogel	VEGF	HUVEC cells; Myocardial infarcted rat model	Gene delivery, Vasculogenesis; cardiac repair	[56]
GO	Bone morphogenetic protein, substance P	Mesenchymal stem cells	Bone regeneration	[57]
PEG-NGS	–	4T1 tumor mice model	Photothermal therapy	[61]
GO-IONPs-Au-PEG	–	Murine breast cancer 4T1 cells; KB cells; BALB/c mice (4T1 murine breast tumor model)	Photothermal treatment, magnetic resonance and X-ray dual-modal imaging	[62]
BSA-RGO	Doxorubicin	U87MG cells	Photoinduced drug release; anticancer treatment	[65]
Porphyrin-GO	–	U87-MG cells	Photothermal therapy; brain cancer treatment	[67]
Aptamer-magnetic GO	Indocyanine green	CCRF-CEM	Photothermal, photodynamic therapy	[70]
GO-fullerene C60	–	HeLa cells	Photothermal, photodynamic therapy	[72]
RGO-Ru-PEG	Ru(II)–polypyridyl complex	A549 cells	Multifunctional imaging and phototherapy	[73]
NGO-UCNP-Ce6	Ce6	HeLa cells; U14 tumor bearing mice	Up-conversion luminescence imaging and PDT/PTT	[76]
Polypyrrole nitrogen-doped few-layer graphene (PPy-NDFLG)	Anti-VEGF	–	VEGF detection	[96]
GO-Antibody	Anti-PSA	–	Prostrate cancer biomarker detection	[101]
GO	Tetramethylrhodamine-labeled LPS-binding peptides	*E. coli*	Sensor for LPS detection	[102]
PABA-GQDs	–	*E. coli*	Bacterial endotoxin detection	[104]
GO	cisplatin	CT26 colorectal carcinoma cells	Imaging and drug delivery	[105]
Carboxyfluorescein-GO nanosheets	ATP	JB6 cells	Molecular probing	[108]
Aminodextran-coated Fe_3_O_4_ nanoparticles-GO		HeLa cell line	Magnetic resonance imaging	[109]
Graphene nanosheets	–	*E. coli* *S. typhimurium* *E. faecalis* *B. subtilis*	Antibacterial activity	[113]
G(IIKK)_4_I-NH_2_-GO	Antimicrobial peptide	*E. coli* *S. aureus*	Antibacterial activity	[115]
Graphene and GO	–	Mesenchymal stem cells	Tissue engineering	[122]
Graphene–chitosan films	–	L929 cells	Scaffold for tissue engineering	[125]
RGO-GelMA hybrid hydrogels	–	Cardiomyocytes	Cardiac tissue engineering	[130]
RGO-silk fibroin	–	Cardiomyocytes	Cardiac tissue engineering	[131]

*NGO* Nanographene oxide, *PEI* polyethylenimine, *NGS* nanographene sheets, *IONPs* iron oxide nanoparticles, *BSA* bovine serum albumin, *UCNP* up-conversion nanoparticles, *PABA* para-aminobenzoic acid

## Importance of Protein Corona

**Fig. 7:**
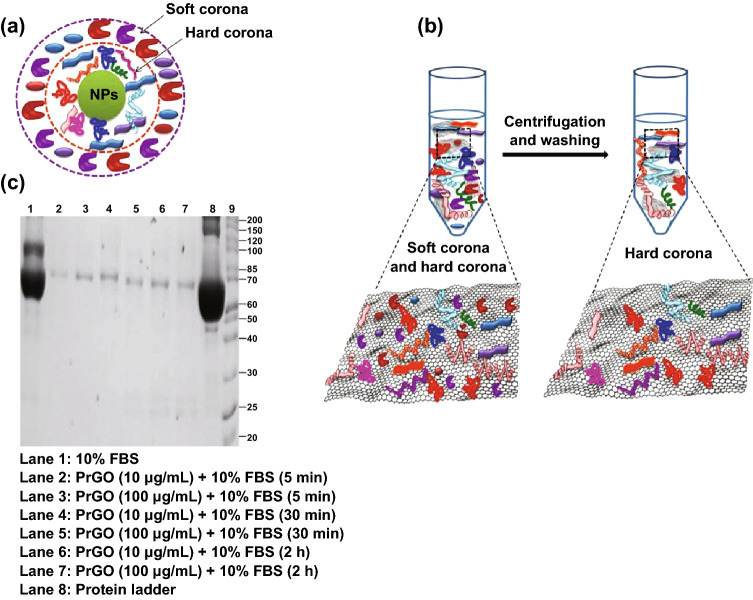
Protein corona. **a** Formation of a hard and a soft corona on the surface of nanoparticles, following the incubation of nanoparticles with serum. **b** Isolation and separation of hard corona proteins that bind to graphene sheets by repeated centrifugation and washing. **c** SDS-PAGE analysis of hard corona proteins isolated from PRGO incubated with FBS at different time periods (5 min, 30 min, and 2 h). Only BSA (~ 66 kDa) formed a hard corona by strongly binding onto the surface of PRGO under different reaction conditions

When a nanoparticle is injected into the body, it enters the circulatory system immediately. The biomolecules present in the blood stream, especially the protein, are quickly adsorbed onto the nanoparticles and form a complex called “corona.” The formation of protein corona alters the physicochemical properties of nanoparticles and modulates the way they interact with the biological system and defines the biological fate of the nanomaterial. Generally, protein corona formed on the surface of nanoparticles is categorized into two layers: “hard” and “soft” corona (Fig. 7a). Hard corona proteins strongly adhere onto the nanoparticle surface, whereas soft corona proteins are loosely bound and are later replaced by strong-affinity proteins (Fig. 7b). Several studies have reported that binding of a protein onto the nanoparticle surface greatly reduces its toxicity. The most abundant proteins such as BSA, immunoglobulin, transferrin, and bovine fibrinogen are found to bind onto the surface of nanoparticles.

Molecular dynamic simulation studies showed that basic amino acid residues (arginine and lysine) and aromatic residues (tryptophan, tyrosine, and phenylalanine) play a key role in binding the protein onto the surface of graphene sheets [140]. The strong *π*–*π* stacking interaction between the graphene sp^2^ carbon atoms and the aromatic rings of amino acids facilitates the adsorption of aromatic residues, whereas the van der Waals interaction between the side chain (guanidinium group) of arginine and the graphene drives the adsorption of basic amino acid residues. During the adsorption process, water molecules are displaced in the interactive region and are squeezed out. The other side of the protein is fully solvated by water. Adsorption also induces large conformational changes in the structure of the protein; hence, the amino acids present in the interior of native proteins are now exposed to the graphene surface. Both GO and RGO have higher protein adsorption capacity than that of single-walled carbon nanotubes (SWCNTs) owing to their flat exposed surfaces. Between the two, RGO adsorbs less protein owing to the lower availability of oxygen groups. Understanding of adsorption kinetics is important to determine the saturation limits, structural and conformational changes, and difference in biological responses. In general, binding of proteins onto the surface of graphene increases with time, and once it reaches the equilibrium, no further adsorption occurs or the protein may fall off from the surface of the nanomaterials. The binding affinity of four proteins (BFG, Ig, Tf, and BSA) on the surface of pristine graphene, GO, and RGO was studied by Chong et al. [141]. Measurement using an Octet RED96 surface plasmon resonance system and AFM analysis revealed that the adsorption capacity increased in the order of BFG > Ig > Tf > BSA. When BSA binds to graphene, it forms complex aggregates with an associated decrease in the α-helical structure and an increase in the β-sheet formation. No further structural changes were noticed with prolonged incubation. On the other hand, binding of BFG onto graphene surfaces induces structural re-arrangement and unfolding of the protein, which exposes buried residues. These buried aromatic amino acid residues (tyrosine, tryptophan, and phenylalanine) strengthen the association between protein and graphene through hydrophobic interactions and *π*–*π* stacking.

Binding of proteins from serum not only increases the size of the nanoparticles but also attenuates the cytotoxicity induced by graphene. The high surface-to-volume ratio of graphene helps in the adsorption of large amounts of proteins. The defects present on the planar surface of graphene provide an additional binding site for proteins. About 90% cell viability was obtained when GO (100 μg mL^−1^) was pre-incubated with fetal bovine serum (FBS) before exposing it to cells [142]. However, this study [142] stated that binding of proteins hinders the direct interaction of GO with cells, which was responsible for the decreased cytotoxicity observed. The molecular dynamic simulation study by Duan et al. [143] further confirmed the above findings. It was explained that the adsorption of the protein (BSA) reduces the interaction between the phospholipid layer and the graphene by decreasing the available surface area as well as by steric hindrance. This, in turn, prevents the extraction of lipids from phospholipid bilayer and the insertion of graphene into the cell membrane.

The major disadvantage of protein corona formation in nanoparticles intended for biomedical applications is the binding of opsonin proteins from the blood stream. Opsonization increases the phagocytosis of nanoparticles by macrophages, thereby reducing their blood half-life and promoting their elimination. Hence, surface functionalization using PEG is commonly followed to reduce protein adsorption and to increase the blood circulation time. It was found that only BSA firmly attaches onto the surface of PEG-functionalized RGO (PRGO) (Fig. 7c). All other proteins are loosely attached and are washed away during centrifugation, forming a soft corona. The concentration of BSA present in FBS is much higher than those of other proteins. Hence, it can effectively bind onto the surface of PRGO and form a hard corona. BSA binding may modulate the uptake and intracellular localization of PRGO. It was already reported in the literature that BSA and SWCNTs coupled with BSA enter into the cells by endocytosis and are primarily localized in lysosomes [144].

## Adverse Effect of Graphene Derivatives

It is well known that the complex interaction between graphene and the biological system induces numerous responses inside the cells. Several reports are currently available on the toxicity induced by pristine graphene and graphene derivatives [145, 146]. These studies maintain that graphene materials should undergo extensive toxicity evaluation before marketing them for any medical purposes. There are several physicochemical factors that influence the consequences of graphene interaction with mammalian cells. Among them, size, shape, lateral dimension, surface chemistry, presence of impurities, and agglomeration are critical for nano-bio interaction.

It is a fact that nanomaterials are often contaminated with endotoxin during synthesis, and handling that may cause septic shock in patients following administration. Endotoxins are lipopolysaccharides derived from the outer membrane of Gram-negative bacteria. LPS binds to pattern recognition receptors and activates immune cells to secrete pro-inflammatory mediators such as tumor necrosis factor (TNF)-α and interleukin (IL)-1β, mediating an inflammatory response. Endotoxin detection is performed using rabbit pyrogen test and LAL assay. Endotoxin detection is difficult in carbon nanomaterials including graphene owing to their interference with LAL assay, which may lead to flawed results. Recently, a TNF-α expression test was developed to detect LPS in graphene samples at noncytotoxic doses [147]. This test is based on the detection of TNF-α secretion in primary human monocyte-derived macrophages incubated in the presence or absence of a specific endotoxin inhibitor. In another study, both gel-clot LAL and chromogenic-based LAL assays were used to detect endotoxins of pristine graphene and multi-wall carbon nanotubes [148]. Repeated autoclaving (three cycles) was done to depyrogenate carbon-based materials. The uptake and pattern of gene expression seemed to be distinct in macrophages exposed to pyrogenated and depyrogenated carbon-based materials.

**Fig. 8:**
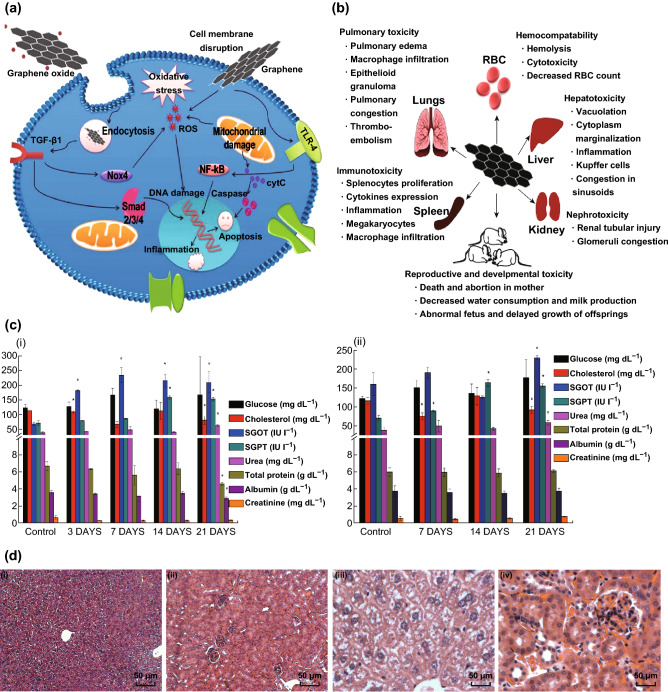
Graphene–bio interaction. **a** Mechanism of graphene nanomaterial-induced toxicity (oxidative stress, apoptosis, and inflammation) in mammalian cells. **b** In vivo organ toxicity induced in animals following graphene administration. **c** Biochemical parameters following single (i) and repeated (ii) administration of PRGO in mice. **d** Histopathological sections of the liver (i—control, iii—PRGO group) and the kidney (ii—control, iv—PRGO group)

The physical interaction between graphene and a cell membrane is considered to be the main mechanism of graphene-induced toxicity. The sharp edges of a graphene sheet cause damage on the cell membrane, resulting in leakage of the intracellular contents. In addition, both GO and RGO are known to induce cytotoxicity, oxidative stress, and DNA damage in mammalian cells. GO promotes nicotinamide adenine dinucleotide phosphate oxidase-dependent ROS formation coupled with deregulation of antioxidant genes, whereas physical stress induced by the presence of RGO results in increased ROS production [149]. Li et al. [145] reported that pristine graphene provokes cytotoxicity by disrupting mitochondrial membrane potential and activates mitochondria-mediated apoptosis. The mitochondrial pathway seems to induce apoptosis by activating MAPKs and the TGF-β signaling pathway. *Bim* and *Bax* (pro-apoptotic factors) activation induces mitochondrial permeabilization, and these factors are released into the cytosol, which eventually activates several cascades of caspase enzymes, ultimately resulting in cell death (Fig. 8a). Another research group suggested that graphene arrests cell-cycle progression and activates apoptosis via the ERK signaling pathway [150]. Graphene also directly disrupts the mitochondrial ETC by acting as an electron acceptor. The disruption of ETC subsequently decreases ATP production, leading to cell death by starvation. Graphene also disrupts actin filaments and destabilizes cytoskeletal organization in cells, leading to decreased cell adhesion and retarded cell migration. GO has been reported to dismantle cytoskeletal organization in cells without affecting the viability [151]. The distribution of *π* electrons in GO absorbs actin monomers and rearranges its secondary structure, creating an interstrand gap. The formation of an interstrand gap in actin tetramers dissociates and cuts them into dimmers. GO can bind actin monomers in a very short period, and the subsequent structural changes become irreversible. Since cell migration is an important phase during cancer progression, the above findings put forth the use of GO for cancer therapy by targeting and disrupting the actin filaments.

Following phagocytosis, graphene initiates a cascade of events that elicit inflammatory responses. Several inflammatory cytokines such as interleukin-6 (IL-6), IL-10, IL-12, TNF-α, monocyte chemotactic protein-1, and interferon-γ are believed to be released upon GO exposure. Graphene activates the TLR and NF-κB signaling pathways, resulting in an inflammatory response (Fig. 8a). GO is also known to promote autophagy by activating the TLR-associated pathway [152]. Upregulation of TLR 4 and TLR 9 activates MyD88 and TRAF-6, inducing the formation of autophagosomes. Moreover, the cells treated with GO showed increased expression of bectin-1 and LC3-II (an autophagic marker).Very few studies have reported on the genotoxic effects of graphene. Wang et al. [153] reported that GO induces genotoxicity in a dose-dependent manner. Graphene effectively interacts with the genomic DNA and induces mutation such as base transitions and deletions. Exposure to GO increases the expression of DNA repair genes such as *ATM* and *Rad1*, which further confirms the mutagenic potential of GO.

The in vitro hemocompatibility of GO and graphene sheets was studied in isolated RBC membranes using hemolysis assay [154]. The results showed GO-induced RBC membrane disruption, which was apparent from the increase in free hemoglobin, whereas very lower hemolytic activity was found in blood samples exposed to graphene sheets. The increase in hemolytic activity of GO is attributed to the strong interaction between the negatively charged GO and the positively charged phosphatidylcholine in RBC membranes. However, because of their hydrophobic nature, graphene sheets form aggregates, resulting in less interaction with the RBC membrane.

GQDs, which have a chemical structure similar to that of graphene but smaller in size, are generally considered to be nontoxic. At a lower concentration (< 50 µg mL^−1^), no obvious cytotoxicity was noticed for GQDs. However, at a concentration of 100 µg mL^−1^, only 50% of the cells were found to be alive. Even after surface functionalization, significant toxicity of GQDs has been reported by a few studies. Like other graphene materials, GQDs also provoke cytotoxicity, increased ROS formation, and genotoxicity in mammalian cells. They can induce DNA damage in cells without affecting their viability by increasing the expression of p53, Rad 51, and OGG1. Although GQDs are mainly distributed in the cytoplasm, with no direct contact with the nucleus, they induce DNA damage indirectly through ROS generation [155]. They have also been reported to induce autophagy in U251 human glioma cells by increasing ROS production. Under excitation, GQD-treated cells exhibit both apoptotic and autophagy characteristics such as phosphatidylserine externalization, caspase activation, DNA damage, formation of autophagic vacuoles, LC3 conversion, and degradation of p62 in autophagic proteolysis [156]. Biological assays such as the Ames test, comet assay, DNA fragmentation assay, and cell-cycle arrest study will help us to understand the mechanism of DNA damage caused by GQDs. Similarly, hydroxylated GQDs (OH-GQDs) induce cell senescence in cancer cells by increasing ROS formation, G0-G1 arrest, and activation of p21, and by inhibiting the phosphorylation of Rb [157]. p21 is an important factor that inhibits cyclin-dependent kinase, which, in turn, inhibits the phosphorylation of Rb, thereby intervening in cell-cycle progression. The cytotoxic effect of OH-GQDs on A549 and H1299 cells revealed that they inhibit cell growth and proliferation by enhancing the accumulation of p53 (tumor suppressor) in the nucleus. The translocated p53 gets activated and binds to DNA, where it acts either as an activator or as a repressor of a specific gene involved in cell cycle, apoptosis, and cellular senescence. However, the role of hydroxyl groups in inducing the localization of p53 is yet to be established. The major concerns with GQDs are related to their potential long-term toxicity and unsatisfactory tumor-targeting efficacy, which can be overcome by surface functionalization using biocompatible polymers and targeting ligands.

Several in vivo reports are available on graphene material toxicity. However, the results are inconsistent because of the difference in the route of exposure and the characteristics of the graphene synthesized. Quantitative measurement of ^14^C-labeled few-layer graphene (FLG) showed that around 47% of the FLG remains in the lungs even 4 weeks post-intratracheal instillation [158]. The presence of FLG was also noticed in the large intestine, small intestine, and stomach. However, in the brain, heart, kidney, muscle, blood, and testis, it was below the detection limit. FLG was cleared from the lungs either by mucociliary clearance or by alveolar macrophages toward the larynx. FLG may pass through the air-blood barrier, enters the blood, and distribute to the liver and spleen. Pulmonary edema, inflammatory cell infiltration, and increase in bronchoalveolar lavage fluid, total protein, and lactate dehydrogenase level were noticed. Figure 8b shows the toxic response initiated in major organs upon graphene exposure.

Recently, the biodistribution and toxicokinetics of PRGO in mice following intraperitoneal and intravenous administration were examined [159]. It was observed that PRGO was effectively absorbed from the systemic circulation as well as from the peritoneal cavity and was distributed in major organs such as the liver, kidney, brain, bone marrow, and spleen. PRGO induced liver obstruction, which was evident from serum biochemistry values (SGOT and SGPT) (Fig. 8c) and from the histological analysis, in which congestion was noticed in both the kidney and the liver (Fig. 8d). Increased immune response was obvious during the initial days of exposure. Although the presence of PRGO was evident in the brain, no pathological lesions were noticed. A very small amount of PRGO was excreted via urine, and the presence of PRGO was evident inside the body even after 21 days of exposure. The functionalization of graphene surface to improve aqueous solubility should be thoroughly investigated to reduce bioaccumulation and to increase the rate of elimination from the body. Spatial and temporal distribution of RGO in the brain was noticed following systemic injection [160]. High concentration of the injected RGO was distributed in the thalamus and hippocampus. RGO enters from the peripheral circulation to the brain by disrupting the blood–brain barrier (BBB) integrity. RGO induces transient and reversible changes in BBB permeability by downregulating the expression of tight junction proteins (occludin, β-catenin, and laminin). However, after 7 days of injection, BBB integrity was regained, suggesting the clearance of RGO from the brain. This study [160] highlights the advantage of using RGO as a noninvasive approach to deliver drug molecules to the brain without compromising their structure and function.

The effect of RGO on female reproductive ability and offspring health was recently studied [161]. In their study, the authors intravenously injected female mice with small-sized RGO (20-150 nm) and larger-sized RGO (200-1500 nm) at three different doses (6.25, 12.5, and 25 mg kg^−1^ body weight) 1 and 30 days before cohabitation. No change in mating behavior was noticed in the female mice. Moreover, less-deformed fetuses were seen in mice injected with RGO at the early stage of gestation (~ 6 days). However, when RGO was injected at a later stage of gestation (~ 20 days), most females died at high dose, whereas the surviving females had abortions. This study [161] also explained that RGO does not cross the placenta nor affects the fetus. However, RGO harms the mother’s health and immune system, increasing its susceptibility to infection, thereby resulting in abnormal fetuses. Hence, this study suggests that the use of RGO for clinical applications during pregnancy is not safe. Table 3 shows the toxic effects exerted by the graphene family of materials.

**Table 3:** Toxic effects of different graphene-derived nanomaterials

Graphene materials	Characteristics	Cells/animals	Dose	Effects	Refs.
Pristine graphene	Thickness: 2–3 nm; Size: 500–1000 nm	RAW 264.7 macrophages	100 µg mL^−1^	Cytotoxic, apoptosis- MAPKs and TGF-beta-pathway	[145]
Pristine graphene;	Thickness: 0.8 nm	Vero cells	0–300 µg mL^−1^	Cytoskeletal re-arrangement; intracellular ROS	[146]
Carboxyl functionalized graphene				Negligible effects on cell viability	
GO	Thickness: 1.0–1.2 nm	RAW264.7 macrophages	100 μg mL^−1^	Toll-like receptor-mediated inflammatory response; Autophagy	[152]
GO	Hydrodynamic diameter: 342 ± 17 nm	RBCs	3.125–200 μg mL^−1^	Hemolysis	[154]
GO-chitosan			100 μg mL^−1^	No hemolysis	
^14^C labeled FLG	Thickness: 0.97–3.94 nm	Mice	0.1 mg mL^−1^ (intratracheal instillation)	Pulmonary toxicity	[158]
RGO	Size: 20–150 nm; 200–1500 nm	Mice	6.25,12.5 and 25 mg kg^−1^ body weight	Deformed foetuses and abortions	[161]
GO	0.2–5 μm	Human platelets;	2 μg mL^−1^	Platelet aggregation;	[162]
		Swiss male mice	250 μg kg^−1^ body weight	Extensive pulmonary thromboembolism	
GO	Lateral dimension: 2 µm and 350 nm	J774A.1, LLC, MCF-7, HepG2, and HUVEC; C57BL/6 male mice	0–20 μg mL^−1^	Cytokines release; Inflammatory response	[163]

*FLG* Few-layered graphene

## Conclusions and Perspectives

The 2D graphene structure offers several advantages over conventional nanoparticles owing to its unique physicochemical characteristics. The successful application of graphene materials will pave a new way for building a nanoplatform in biomedical research. However, the use of graphene in the biomedical field is still in its nascent stage, with numerous challenges to be overcome. The toxicity induced by graphene nanomaterials in a biological system causes significant safety concerns pertaining to the use of the nanoparticles. Several measures to reduce their toxicity such as the use of biocompatible polymers and a green route for synthesis have already been reported in the literature. Desirable characteristics can be obtained by manipulating the synthesis under controlled conditions. More attention should be paid to understand the nano-bio interaction of graphene materials in a living system. A simple cytotoxicity test is not a valid screening criterion to describe any material as being biocompatible and suitable for clinical translation. Graphene is included in the list of hazardous materials by the European Scientific Committee on Emerging and Newly Identified Health Risks. There are still many gaps to be filled, considering the toxic potential of graphene. All physicochemical parameters including the size, shape, agglomeration, layer thickness, lateral dimension, and atomic composition should be considered while evaluating the toxicity. From a biological perspective, the effect of concentration, duration, route of exposure, and presence of impurities should be thoroughly investigated. The contradictory outcomes, difference in synthesis methods, and lack of reproducibility hinder the use of graphene in real-world applications. Since there are no regulatory guidelines available for testing the toxicity of nanomaterials, a standard protocol must be established to avoid such conflicts. In conclusion, graphene promises an exciting nanoplatform for biomedical applications, yet there are many questions that need to be addressed. It is recommended that graphene derivatives should undergo extensive safety evaluations or validations before considering them safe for biomedical applications or clinical use.
